# Type I Non-ST Segment Elevation Myocardial Infarction (NSTEMI) Followed by Type II in a Young Patient With Fibromuscular Dysplasia (FMD) Presented With Hypertensive Emergency: A Case Report

**DOI:** 10.7759/cureus.40401

**Published:** 2023-06-14

**Authors:** Mubariz A Hassan, John Gharbin, Siddharth Bajaj, Ahmed Brgdar

**Affiliations:** 1 Internal Medicine, Howard University Hospital, Washington, DC, USA

**Keywords:** severe coronary artery disease, cardiac cath, renovascular hypertension, non-st segment elevation myocardial infarction (nstemi), renal fibromuscular dysplasia

## Abstract

This article presents a case report highlighting the association between fibromuscular dysplasia (FMD) and acute myocardial infarction in a 25-year-old female patient with multiple cardiovascular comorbidities. Initially presenting with a hypertensive emergency, the patient subsequently developed acute coronary syndrome. MRI revealed irregular narrowing of the bilateral renal arteries, consistent with a diagnosis of FMD. Further evaluation through cardiac catheterization confirmed 95% stenosis of the mid-circumflex artery, necessitating percutaneous coronary intervention (PCI).

Fibromuscular dysplasia has been frequently reported in conjunction with coronary artery dissection leading to acute coronary syndrome, especially in young females. Here, we describe the case of FMD without any coronary artery dissection. The presence of FMD highlights the need for comprehensive evaluation and management in patients with multiple cardiovascular risk factors.

The recognition of FMD as an underlying pathology in acute myocardial infarction is crucial for appropriate intervention strategies. In this particular case, PCI was successfully performed to address the significant stenosis of the mid-circumflex artery. These findings emphasize the importance of considering FMD as a potential contributing factor in young patients presenting with acute coronary syndrome, particularly in the context of renal artery involvement. Increased awareness among healthcare providers regarding the association between FMD and acute myocardial infarction can aid in prompt diagnosis, appropriate management, and improved patient outcomes.

## Introduction

This article presents a case report highlighting the association between FMD and acute myocardial infarction in a 25-year-old female patient with multiple cardiovascular comorbidities. Initially presenting with a hypertensive emergency, the patient subsequently developed acute coronary syndrome. MRI revealed irregular narrowing of the bilateral renal arteries, consistent with a diagnosis of FMD. Further evaluation through cardiac catheterization confirmed 95% stenosis of the mid-circumflex artery, necessitating percutaneous coronary intervention (PCI).

Fibromuscular dysplasia has been frequently reported in conjunction with coronary artery dissection leading to acute coronary syndrome, especially in young females [1]. Here, we describe the case of FMD without any coronary artery dissection. The presence of FMD highlights the need for comprehensive evaluation and management in patients with multiple cardiovascular risk factors.

The recognition of FMD as an underlying pathology in acute myocardial infarction is crucial for appropriate intervention strategies. In this particular case, PCI was successfully performed to address the significant stenosis of the mid-circumflex artery. These findings emphasize the importance of considering FMD as a potential contributing factor in young patients presenting with acute coronary syndrome, particularly in the context of renal artery involvement. Increased awareness among healthcare providers regarding the association between FMD and acute myocardial infarction can aid in prompt diagnosis, appropriate management, and improved patient outcomes.

## Case presentation

The case revolves around a 25-year-old patient with a complex medical history. The individual had uncontrolled diabetes mellitus, severe obesity, and uncontrolled hypertension, with no prior use of antihypertensive medications. The patient presented to the ophthalmology clinic due to a hypertensive emergency, which was further complicated by Grade IV hypertensive retinopathy. The patient's main complaint was experiencing intermittent flutters in the right visual field over the past few weeks, prompting the visit to the eye clinic. Upon examination in the emergency department, the patient exhibited Grade 3 disc edema accompanied by cotton wool spots, scattered hemorrhages, and tortuous vessels. The blood pressure reading revealed severe hypertension at 190/100 mmHg. Subsequent investigations revealed elevated creatinine levels along with elevated cardiac biomarkers as shown in Table 1. Consequently, the patient was admitted to the medical intensive care unit (MICU) for the management of the hypertensive emergency. During the hospital stay, the patient also underwent extensive autoimmune workup that came back negative for any inciting etiology. Thyroid function, cortisol levels, metanephrines, and nor-metanephrines levels were normal. Further blood work for apolipoprotein B and lipoprotein A also came back within normal range along with serum aldosterone and plasma renin activity.

**Table 1:** Laboratory results. BNP, brain natriuretic peptide

Basic labs	Results	Reference range
White blood cells	10.79	3.2-10.6 x 10^9^/L
Hemoglobin	9.1	14.6-17.8 g/dL
Hematocrit	26.6	40.8%-51.9%
Platelets	526	177-406 x 10^9^/L
Sodium	141	135-145 mEq/L
Chloride	109	95-111 mEq/L
Blood urea nitrogen	38	7-25 mg/dL
Creatinine	2.59	0.6-1.2 mg/dL
Potassium	5.0	3.5-5.1 mEq//L
Magnesium	1.90	1.7-2.5 mg/dL
BNP	1077	<100 pg/mL
Troponin	3.12 > 3.08 > 6.46	<0.03 ng/mL

**Figure 1:**
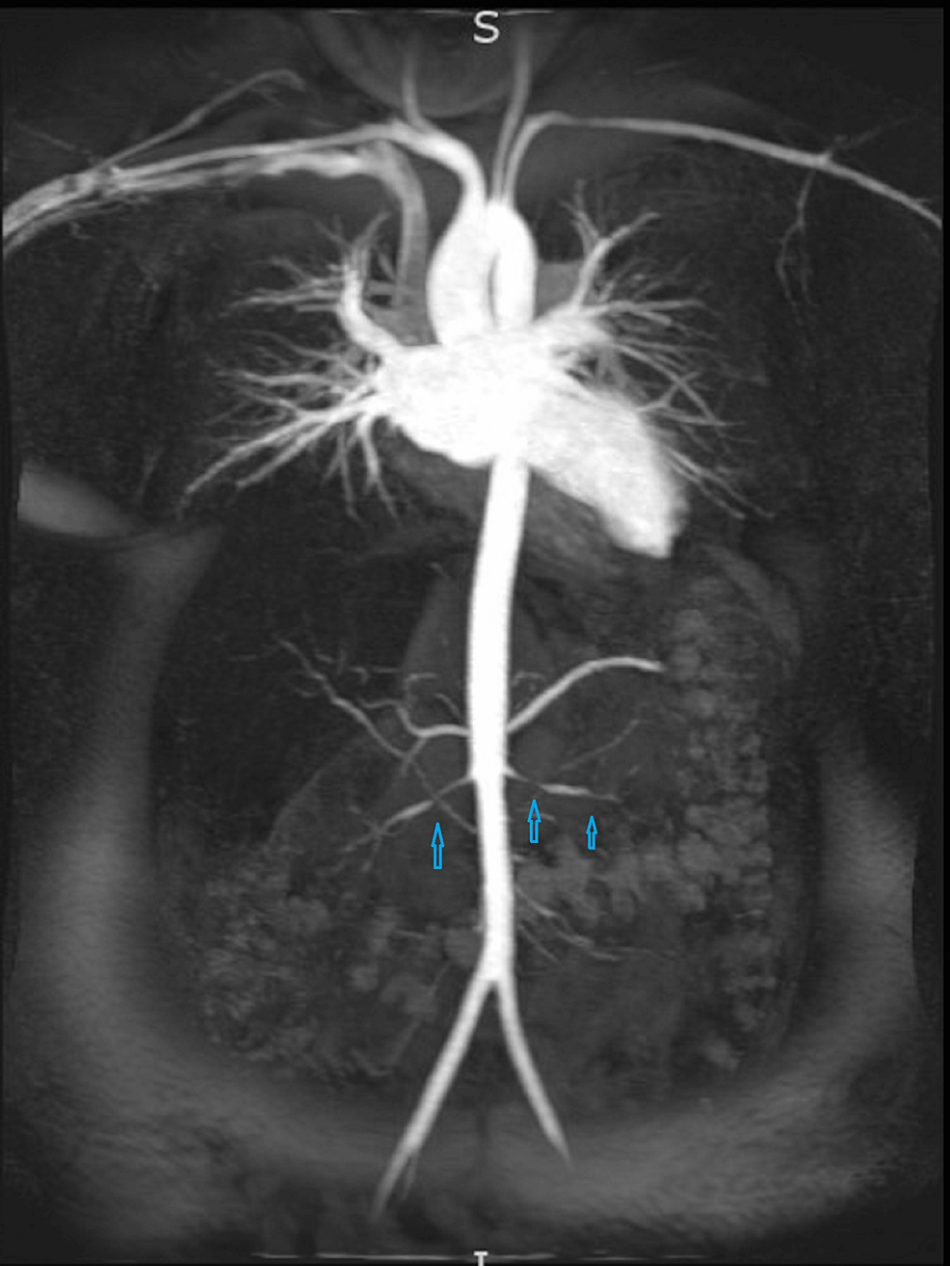
MRI showing FMD of renal arteries as shown by arrows. FMD, fibromuscular dysplasia

The cardiology team was consulted to evaluate the patient for cardiomyopathy. The initial echocardiogram revealed an ejection fraction of 25%-30% with globally reduced wall motion, while blood pressure control remained poor. However, with an improvement in blood pressure, a repeat cardiac echocardiogram demonstrated improved systolic function. Further imaging studies, including an abdominal and pelvic MRI, was done that ruled out any dissections or aneurysms but revealed irregular narrowing of the bilateral renal arteries consistent with FMD that can be seen in Figure 1.

**Figure 2:**
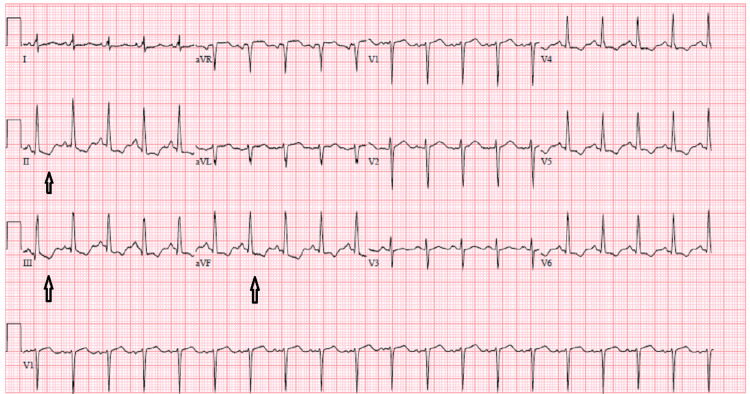
ECG showing sinus tachycardia with ST-segment changes representing possible sub-endocardial injury as shown with arrows. ECG, electrocardiogram

During the patient's hospital stay, an abrupt onset of substernal chest pain occurred, accompanied by a sudden rise in troponin levels to 6.46 ng/dL. Electrocardiogram (ECG) was done that showed concerning changes for possible inferior sub-endocardial injury as shown in Figure 2. Urgent intervention was planned, and the patient was taken to the cardiac catheterization lab. The procedure revealed 95% stenosis of the mid-circumflex artery, necessitating PCI with the placement of an intravenous ultrasound-guided drug-eluting stent (IVUS DES). Subsequently, the patient was initiated on aspirin, prasugrel, and atorvastatin for secondary prevention. With stabilization achieved, the patient was discharged home and scheduled for a close follow-up in the outpatient setting.

## Discussion

Fibromuscular dysplasia is a vascular disease that affects the arteries and is seen more commonly in women [2]. It mainly involves medium-sized arteries and is different from atherosclerosis or plaque buildup within the arteries [3]. Generally, we can distinguish the artery blockage of FMD from those of atherosclerosis or plaque based on the appearance or its location. Blockage due to atherosclerosis or plaque buildup mostly develops at a branch point in an artery, whereas blockage due to FMD tends to develop further along the vessel [3]. FMD results from abnormal development of the arterial cell wall particularly involving the media part and is less likely to involve the vessel intima. Multifocal type is the most common variant known which affects the arteries resembling strings of beads due to alternating areas of stenosis and dilatations [4]. FMD can also cause singular stenosis (focal type) and is commonly associated with arterial tortuosity, dissections, or aneurysms. The exact underlying mechanism for the pathogenesis of FMD is not fully known but it has been related to environmental and genetic factors. The most common association of FMD has been seen in smokers and female populations [4]. Almost 80%-90% of patients with FMD are women, although men can also develop the disease and have an aggressive course with a higher frequency of aneurysms and dissections [5].

Here, we would also like to briefly mention about two of the several types of non-ST segment elevation myocardial infarction (NSTEMI). Type 1 NSTEMI refers to a non-ST Segment elevation myocardial infarction caused by a rupture or erosion of a coronary artery plaque, leading to partial or complete occlusion of the artery. It is typically associated with atherosclerosis and is characterized by the release of cardiac biomarkers, such as troponin, indicating myocardial damage. On the other hand, Type 2 NSTEMI is a non-ST segment elevation myocardial infarction resulting from an imbalance between myocardial oxygen supply and demand, usually due to factors other than coronary artery plaque rupture. Common causes include severe coronary artery vasospasm, coronary microvascular dysfunction, severe anemia, hypotension, uncontrolled hypertension, or tachyarrhythmias [5]. It is important to identify and address the underlying cause to optimize patient management and outcomes.

Based on our literature search and case reports, FMD is mostly associated with coronary artery dissection leading to acute coronary syndrome [6]. Although FMD is highly prevalent in patients with spontaneous coronary artery dissection (SCAD), coronary dissection is an uncommon occurrence among patients with FMD [6]. FMD is a nonatherosclerotic vascular disease and the most commonly affected vascular beds are renal and carotid arteries. The exact frequency of FMD of coronary arteries is still not well known since the appearance of a string of beads is rarely seen. In most cases, we see distal tapering, non-atherosclerotic stenosis, or dissection of the artery [6]. The majority of patients with coronary artery manifestations of FMD present with dissection of an epicardial artery (left anterior descending, circumflex, or right coronary artery) or any major branch which can clinically lead to acute myocardial infarction, unstable angina, left ventricular dysfunction, or even sudden cardiac death [7].

The manifestations of noncoronary FMD depend on the involvement of the arterial beds, most often with renal artery FMD, along with uncontrolled hypertension as seen in our patient. Optical coherence tomography (OCT) and intravascular ultrasound (IVUS) along with angiography are among the modalities that are helpful for diagnostic purposes [8]. Outcomes are variables depending on organ involvement and range from aneurysm rupture, dissection, and or infarction [9].

## Conclusions

Fibromuscular dysplasia remains an underrecognized condition, particularly in the coronary arteries, and is more prevalent in women. Its exact underlying mechanism is not fully understood, but environmental and genetic factors are believed to play a role. Prompt diagnosis and management are crucial to prevent complications such as aneurysm rupture, dissection, or infarction. Further research and awareness are necessary to better understand the clinical manifestations, optimal diagnostic modalities, and long-term outcomes associated with FMD in various vascular beds. The occurrence of hypertensive emergency, hypertensive retinopathy, renal artery FMD, and acute coronary syndrome highlights the need for a comprehensive evaluation, prompt intervention, and a multidisciplinary approach to optimize patient outcomes. Close monitoring and follow-up will be crucial in managing such patients' long-term cardiovascular health.
